# Dysregulated Functions of Lung Macrophage Populations in COPD

**DOI:** 10.1155/2018/2349045

**Published:** 2018-02-18

**Authors:** Theodore S. Kapellos, Kevin Bassler, Anna C. Aschenbrenner, Wataru Fujii, Joachim L. Schultze

**Affiliations:** ^1^Genomics & Immunoregulation, Life and Medical Sciences Institute (LIMES), Carl-Troll-Str. 31, 53115 Bonn, Germany; ^2^Platform for Single Cell Genomics and Epigenomics, German Center for Neurodegenerative Diseases and University of Bonn, Sigmund-Freud-Str. 27, 53175 Bonn, Germany

## Abstract

Chronic obstructive pulmonary disease (COPD) is a diverse respiratory disease characterised by bronchiolitis, small airway obstruction, and emphysema. Innate immune cells play a pivotal role in the disease's progression, and in particular, lung macrophages exploit their prevalence and strategic localisation to orchestrate immune responses. To date, alveolar and interstitial resident macrophages as well as blood monocytes have been described in the lungs of patients with COPD contributing to disease pathology by changes in their functional repertoire. In this review, we summarise recent evidence from human studies and work with animal models of COPD with regard to altered functions of each of these myeloid cell populations. We primarily focus on the dysregulated capacity of alveolar macrophages to secrete proinflammatory mediators and proteases, induce oxidative stress, engulf microbes and apoptotic cells, and express surface and intracellular markers in patients with COPD. In addition, we discuss the differences in the responses between alveolar macrophages and interstitial macrophages/monocytes in the disease and propose how the field should advance to better understand the implications of lung macrophage functions in COPD.

## 1. Lung Macrophage Populations in Mice and Humans

**Figure 1:**
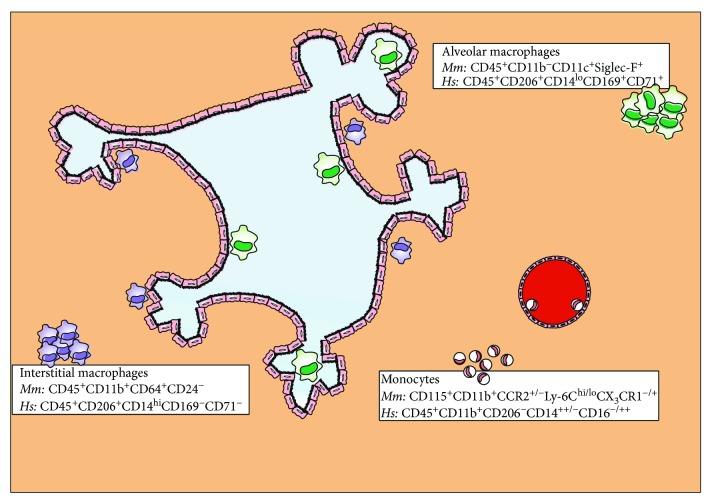
Murine and human lung macrophage populations under steady-state conditions. AMs reside at the airspaces of the lung, while IMs localise in the interstitial space between the alveoli and blood vessels. In both the murine and human lungs, there is also a monocyte population which enters the tissue from blood vessels. AMs are the biggest of all three lung macrophage populations, are potent phagocytes, and secrete a range of proinflammatory mediators. IMs are smaller than AMs but display comparable phagocytic capacity and ability to produce soluble factors. They are believed to serve as an intermediate step in monocyte differentiation towards AMs and demonstrate proliferative potential. Finally, monocytes are sensitive to migratory gradients and have been shown to exhibit proinflammatory mediator capacity, but no antigen presentation. The currently acceptable nomenclatures for AMs, IMs, and monocytes in mice (*Mm*) and humans (*Hs*) are indicated next to each population.

The lung is constantly exposed to the host's outer environment; therefore, constitutively active mechanisms are required to monitor for irritants and infections with pathogens. This pivotal sentinel function is assumed by lung-resident immune cell populations including macrophages, dendritic cells (DCs), and airway epithelial cells [1]. To date, three major myeloid cell populations have been identified in the lung which differ in their exact localisation in the tissue and their developmental origin (Figure 1): resident alveolar macrophages (AMs), resident interstitial macrophages (IMs), and blood monocytes [2–4].

AMs reside in the airspaces of the lung, whereas IMs are found in the interstitial space between the alveoli and blood vessels. Morphological observation of these two populations indicated that AMs are larger in size than IMs [5]. In addition, phenotypic characterisation of AMs and IMs in mice revealed differences in the expression levels of MHC class-II, CD11b, CD14, CD45, CD54, CD68, CD71, CD204, CD206, and Siglec-F [5–9]. Altogether, lung-resident macrophages have been characterized as CD11c^+^CD11b^lo^ cells and can be distinguished from recruited cells during endotoxin or viral-induced inflammation by the level of CD11b expression [10]. In humans, AMs are described as CD45^+^CD206^+^CD14^lo^CD71^+^CD169^+^ cells, whereas IMs are reported as CD45^+^CD206^+^CD14^hi^CD71^−^CD169^−^ cells [11]. However, recently a study suggested high expression of the mannose receptor (CD206) in both macrophage populations and revealed two AM subpopulations with differential expression of the hemoglobin-haptoglobin complex scavenger receptor CD163 [12]. Lastly, Desch et al. found that human AMs (CD206^+^CD14^lo^HLA-DR^+^CD64^+^CD141^+^ cells) could be distinguished from lung tissue monocytes based on CD14 and CD16 surface expression [13].

Functionally, although a small fraction of AMs was shown to be present in lymph nodes in *S. pneumoniae*-infected mice [14], IMs are considered to be classical modulators of adaptive immunity in human and murine lungs [7, 15–18]. In humans and rodents, AMs have been reported to remove surfactants and debris [19], suppress adaptive immunity [20, 21], and regulate neutrophil and monocyte recruitment to the lung [22–24]. With regard to other typical macrophage functions, both populations display high phagocytic capacity [5, 25], but AMs are considered to be more potent phagocytes [17, 26–28] and they were shown to secrete proinflammatory mediators and reactive oxygen species (ROS) upon activation in animal studies [17, 27, 29, 30].

Research on both human and animal AMs challenged the homogeneity of this population [31, 32]. Instead, density-gradient centrifugation splits them into distinct subpopulations with differences in the expression of surface markers and intracellular enzymes as well as tumour lysis, migration, cytotoxicity, phagocytosis, lymphoproliferative response augmentation, soluble mediator release, and procoagulant activity [33–42].

Under steady-state conditions, the replenishment of AMs in humans and mice occurs mainly via self-renewal as recently demonstrated in long-term lung transplant, parabiosis, and fate-mapping studies [43–45]. During lung inflammation, a proportion of AMs dies by apoptosis and the cells are replenished in part by local proliferation of local stem cells, but also via the recruitment of blood mononuclear phagocytes [46–48]. IMs acquire proinflammatory markers upon activation, such as CD40, CD80, and CD86, and their numbers are increased in mice [6]. Between the two populations, AMs secrete more TNF-*α*, but less IL-6, IL-1ra, and IL-10 than IMs in rats [49]. Furthermore, in humans, the two populations exhibit differential sensitivity to pathogen recognition receptor (PRR) activation with IMs being less sensitive to TLR9 priming [5].

IMs are not a homogeneous population either, and in the rat lung interstitium, they are currently believed to be contaminated with up to 20% AMs [50]. Similar to AMs, several density-defined populations have been identified exhibiting differential prostaglandin secretion, migration, and phagocytosis capabilities [51–53]. It has long been considered that IMs are an intermediate step in maturation of infiltrating blood monocytes towards AMs [54, 55] because they display blunt lamellipodia and fewer lamellar inclusions than AMs and are morphologically more closely related to blood monocytes [4, 56, 57]. Moreover, in mice, they seem to proliferate more than AMs [17]. However, considering more recent findings in macrophage ontogeny and the possibility to measure hundreds to thousands of genes at the single cell level, these observations need to be revisited.

Monocytes are divided into subpopulations in both humans and mice (reviewed in [58]). Fate-mapping experiments in mice unraveled a CD115^+^CD11b^+^Ly-6C^hi^CCR2^+^ and a CD115^+^CD11b^+^Ly-6C^lo^ monocyte population [59, 60]. Ly-6C^lo^ monocytes express high levels of the fractalkine receptor CX_3_CR1, and they were shown to crawl inside blood vessels via lymphocyte function-associated antigen 1 interactions with the endothelial lining [60, 61]. Upon activation with an inflammatory stimulus, they rapidly respond by secreting TNF-*α* [62]. In contrast, Ly-6C^hi^CCR2^+^CX_3_CR1^−^GR-1^+^ monocytes are actively recruited to inflamed tissues where they can differentiate into so-called inflammatory DCs or different flavours of macrophages [60, 63–65]. This subset was shown to express high levels of chemokine receptors, complement peptides, and annexins, while Ly-6C^lo^ monocytes express more MHC class-II, growth factors, integrins, and scavenger receptors [66, 67].

In analogy to mice, human monocytes are divided into different subsets including CD14^++^CD16^−^ (classical), CD14^+^CD16^+^ (intermediate), and CD14^−^CD16^+^ (nonclassical) [58]. All subsets are CD206^−^CD64^+^ [13] and express CX_3_CR1 and CXCR4 (CD16^+^ monocytes express CX_3_CR1 at higher levels which allows them to adhere firmly to vessel walls [58]). Classical monocytes also express several CC chemokine receptors [58, 60] and are characterised by an antimicrobial phenotype [68]. Intermediate monocytes express genes related to antigen processing and presentation, transendothelial migration, and angiogenesis and secrete higher amounts of cytokines and ROS than other subsets [68, 69]. Human classical monocytes resemble murine Ly-6C^hi^ monocytes, whereas nonclassical monocytes were described to be the counterparts of Ly-6C^lo^ monocytes (reviewed in [64]). The human blood monocyte population structure was recently challenged by Villani et al. who, by application of single cell RNA sequencing, suggested that peripheral blood monocytes can be further divided in four subsets [70]. Whether this also holds true for lung monocytes awaits further investigation.

## 2. Chronic Obstructive Pulmonary Disease (COPD): Epidemiology, Pathology, and the Role of the Immune System

COPD is a chronic disease of the lower respiratory tract and is characterised by irreversible airway obstruction, chronic bronchitis, and loss of alveolar parenchyma (emphysema) [71]. It affects almost equally men and women, has its onset in midlife, and progresses slowly during adulthood [72] resulting in airway obstruction by mucus exudates and lung tissue remodelling [71]. Patients with COPD are diagnosed as stage 1 (mild) to 4 (very severe) based on spirometric grading as well as group A to D based on clinical assessment of symptoms and exacerbation risk according to GOLD classification [73]. Besides the well-documented increase in patients' disability-adjusted life years, COPD is also a huge economic burden for countries due to its chronic nature, the exacerbations which lead to patient hospitalisation and the lack of effective drugs [74–76].

COPD ranked sixth globally as a leading cause of death in 1990 and is projected to rank third by 2020 accounting for 7% of total deaths worldwide [73, 77, 78]. There are several causative factors for the disease (reviewed in [79, 80]) including environmental factors, such as smoking (which is now accepted as the main causal factor of the disease), the use of biomass fuel, occupational exposure to toxic gases or dust, infections, outdoor pollution, genetic susceptibility as exemplified by the deficiency of *α*_1_-antitrypsin (reviewed in [81]), and accelerated lung ageing [82, 83].

COPD is thought to be initiated when inhaled irritants activate innate immunity either directly by triggering common PRRs on immune and bronchial epithelial cells or indirectly by inducing the release of danger signals by epithelial and endothelial cells [84–86]. In fact, the subsequent recruitment of blood leukocytes and the destruction of lung tissue are TLR-dependent and macrophage activation occurs in an inflammasome-dependent manner [87]. Patients with COPD present with elevated levels of a broad range of proinflammatory mediators in their bronchial lavage, such as TNF-*α*, IL-8, CCL2, CCL3, LTB_4_, myeloperoxidase, and eosinophilic cationic protein among others [88–94]. In parallel, the vasculature upregulates surface adhesion molecules [95] and becomes permeable to attract blood neutrophils, monocytes, and eosinophils to the lung. Secretion of the tissue remodelling cytokine TGF-*β* by epithelial cells has also been reported to relate to small airway obstruction in COPD [96].

Neutrophil percentages in COPD correlate with deterioration of lung function and airway obstruction [97] and, together with macrophages [98], they contribute to disease pathology via the production of extracellular matrix- (ECM-) degrading enzymes [99]. Disintegrated alveolar wall components can be readily detected in the biological fluids of patients with COPD and are significantly higher than in healthy smokers [100]. Neutrophil elastase (NE) and metalloproteinases (MMPs) cause lung tissue destruction and trigger mucus secretion which obstructs small airways [101]. The imbalance between proteases and protease inhibitors in the lungs of patients with COPD causes enhanced chemotactic factor secretion by macrophages and further amplification of neutrophil recruitment [102].

In the healthy lung, DC sample inhaled exogenous material or apoptotic cells to induce immune tolerance or initiate appropriate immune responses [1]. In COPD, DCs accumulate in the lung in an IL-1*α*-dependent manner following a CCL20-CCR6 axis [103, 104]. Recent reports have suggested that the numbers of the various DC subsets are differentially altered in the several lung compartments. For example, Langerhans-type DCs have been observed selectively in small airways [105], whereas the numbers of bronchial mucosal DCs in the epithelium as well as the migratory CD83^+^ and CCR7^+^ DC subsets are reduced in patients with COPD [106, 107]. The dysregulated localisation of these immune cells comes together with altered immune responses regulated by the different subsets [108]; cigarette smoke and the lung inflammatory milieu decrease lung myeloid DC maturation [109, 110] and cause an imbalance to the costimulatory status of these cells [111]. In contrast, CD1c^+^ DCs favour tolerogenic signalling and the induction of regulatory T cells [112].

DC-mediated CD4^+^ T cell activation is predominantly skewed to a T_H_1 phenotype [113], although T_H_17 cells have also been found in the lungs of patients with COPD [114, 115]. However, in the epithelium, submucosa, and adventitia of peripheral airways of patients with COPD, CXCR3-expressing CD8^+^ cells are the predominant T cell subtype [116]. CD8^+^ lymphocytes contribute to tissue injury and cell death in the lung via the release of proteolytic enzymes, such as perforin and granzymes [117–120]. Finally, the numbers of regulatory T cells have been demonstrated to be in decline in patients with COPD in comparison with healthy smokers which highlights another causality factor for the chronicity of the disease [121, 122]. Regarding the factors responsible for the increase in T cell numbers, Di Stefano et al. showed that IL-27 secretion by CD68^+^ cells in the BAL of patients with COPD may contribute to IFN-*γ* and granzyme B secretion by CD8^+^ lymphocytes as well as the induction of regulatory T cells [123]. However, more studies are needed to clarify the role of T cells as part of an efficient acute or a dysregulated chronic response mounted by alterations in innate immunity.

In 2006, the presence of B cells was also described in lymphoid follicles in small airways and lung parenchyma of patients with COPD and animal models [124]. Supporting evidence came from the detection of elevated levels of B cell-activating factor in lymphoid follicles which inversely correlated with lung function [125]. Although the nature of the antigens that activate B cells is not fully known, it has been speculated that they range from cigarette smoke irritants [126] to cell death and ECM degradation by-products, microbial components, and autoantigens [127].

Finally, a frequent manifestation of COPD is the colonisation of the patients' lungs by bacteria and viruses (likely due to impaired phagocytosis by AMs [126]) which cause exacerbations diminishing the patients' quality of life [128, 129]. *H. influenzae*, *S. pneumoniae*, and *M. catarrhalis* are most usually detected in patients with frequent exacerbations, while *P. aeruginosa* infections account for exacerbations in patients with severe COPD [130–132]. Furthermore, in recent years, the role of viral infections in the worsening of patients' health has begun to be appreciated and research has focused on the identification of the immune cells and mechanisms that contribute to the loss of lung function. Rhinoviruses [133], picornavirus [134], adenoviruses, the respiratory syncytial virus, and influenza virus are the most common viruses found in the sputum of patients with COPD and are responsible for about half of all exacerbations observed (reviewed in [135]). Infections augment the innate immune responses and lung tissue remodelling in mice [136], while human patients present with dysregulated neutrophil and T cell mobilisation [89, 137], increased proinflammatory mediator levels [138, 139], and antibacterial humoral responses [140].

## 3. Why the Functions of Lung Macrophage Populations in COPD Warrant Further Investigation

The numbers of lung-resident macrophages in the lung have been reported to be dramatically increased in COPD due to the recruitment of blood leukocytes from the periphery [141, 142]. Macrophages are plastic cells and respond in several ways to accommodate changes in their microenvironment. For example, AMs from smokers present with increased expression of cytokines and chemokines, growth factors, proteases, antioxidant proteins, adhesion molecules, transcription regulators, and signalling pathway genes, whereas they reduce expression of genes related to neutrophil activation, serine protease inhibitors, and macrophage differentiation genes [143]. Consequently, in the constantly changing microenvironment of the COPD lung, resident macrophages will respond accordingly and shape their effector functions to orchestrate the immune responses. Hence, the study of the functions of lung macrophage populations as well as their interplay with other immune cells and the lung stroma has the potential to enhance our understanding of COPD pathology and provide with novel biomarkers and therapeutic targets.

## 4. AMs in COPD

**Figure 2:**
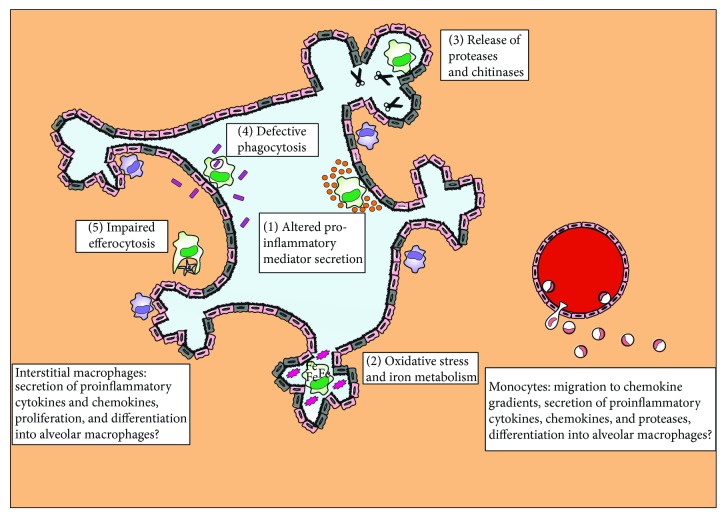
Lung macrophage population functions in COPD. AMs exhibit alterations in their physiological responses in COPD; the secretion of proinflammatory cytokines and chemokines is dysregulated (1). The cells undergo oxidative stress and secrete ROS and nitrite species into the lung micro-environment (2), they store intracellularly large amounts of iron (2), and they overexpress and release proteases which cause alveolar tissue destruction (3). In contrast, processes, such as phagocytosis of microbes (4) and apoptotic neutrophils or epithelial cells (5), are downregulated in AMs from patients with COPD, an observation which could explain the frequent colonisation of the lungs with bacteria and viruses in exacerbations. In the meantime, monocytes are recruited from blood vessels following chemokine gradients and contribute to disease pathology via the secretion of proinflammatory mediators and proteases. It is also believed that monocytes differentiate into macrophages via an intermediate step of IMs which morphologically and functionally resemble monocytes.

Over the last decades, numerous studies have accumulated knowledge about the role and functions of AMs in COPD. Major aspects of change in cellular functions concern the secretion of proinflammatory mediators, the induction of oxidative stress, the deregulation of the protease-protease inhibitor balance, and the impairment of pathogen phagocytosis as well as changes in gene expression which we highlight next (Figure 2 and Table 1). Many of these studies have been performed in the pregenomic era and most of them prior to the era of single cell genomics. Therefore, as for every other field in life sciences, some of the previous findings might be challenged once we have applied cutting-edge technologies to better understand the basic unit of life—the cell—and its changed functionality in complex diseases like COPD. Nevertheless, we review the current knowledge which has often been obtained only at the population level, but not at the single cell level yet.

### 4.1. Altered Secretion of Proinflammatory Mediators

AMs from patients with COPD present with alterations in the secretion of cytokines and chemokines. In particular, the levels of TNF-*α*, IL-1*β*, IL-6, IL-10, IL-12, CCL2, CCL5, CCL7, CCL13, CCL22, IL-8, CXCL9, and CXCL10 in AM secretions from smokers were significantly different from healthy subjects [126, 144–152]. Similarly, the levels of the chemokine receptors CCR2 and CCR5 were found to be increased [153, 154]. Moreover, macrophages primed with endotoxin and cigarette smoke presented with delayed IL-1*β* and IL-6 secretion in comparison with control endotoxin-treated cells and a subsequent increase in IL-8 levels [155]. Finally, sputum macrophages from patients with COPD were found to express more prostaglandin H synthases 1 and 2 than unaffected control subjects [156].

TLR signalling is pivotal for proinflammatory mediator secretion by macrophages in COPD as exemplified by the TLR4-dependent cigarette smoke-mediated activation of human macrophages [157]. Downstream of TLR activation, lung macrophages from patients with COPD also exhibit dysregulated signalling including p38, ERK1/2, JNK and IRAK-1 phosphorylation, I*κ*B*α* expression, and NF-*κ*B p65 activation compared to healthy individuals [145, 147, 155]. Finally, the importance of TLR signalling for macrophage proinflammatory mediator secretion in COPD is also illustrated by the downregulation of the chemokines CXCL9, CXCL10, and CXCL11 [147, 154, 158] as a result of the attenuation of TLR3 activation [158]. While all these findings are very informative, we still do not have an integrative, systemic, and causal model of the main regulatory mechanisms operative in AMs of patients with COPD.

Therefore, more light needs to be shed on the molecular programmers that drive these functional differences and conclude whether these are observed in a fraction of the AM population. To this end, microRNAs have been involved in the regulation of proinflammatory cytokine release by AMs [159], whereas recent investigation into the epigenetic networks active in macrophage populations of patients with COPD and healthy smokers revealed that the histone deacetylases HDAC2 and HDAC3 are downregulated in comparison with healthy individuals and correlate negatively with disease severity [160, 161]. Similarly, Yang et al. showed that oxidative stress induces posttranslational modifications on HDAC2 which are responsible for the loss of function of this enzyme's activity [162]. Taken together, it seems plausible to hypothesise that defects in the transcriptional and epigenetic regulation of proinflammatory genes in COPD cause dysregulated TLR signalling and effector biomolecule secretion by AMs.

### 4.2. Induced Oxidative Stress

Inhaled cigarette smoke and airborne pollutants induce oxidative stress in human lungs. In more detail, cigarette smoke contains approximately 4000 chemicals including oxidants which impact lung physiology [163, 164]. On the contrary, the antioxidant protein glutathione (GSH) is heavily suppressed [150, 165] in macrophages by the actions of aldehydes in cigarette smoke [166] and biomolecules are modified (e.g., protein carbonylation) [147] leading to deleterious effects on living cells.

In response, AMs from patients with COPD have been demonstrated to express the nitrite synthase gene iNOS, but less heme oxygenase 1 (HO-1) than healthy smokers [167]. As mentioned above, other inflammation-related molecules, such as the histone deacetylases HDAC2 and SIRT1, are downregulated in AMs in an oxidative stress-dependent manner [165, 168, 169]. Eventually, cigarette smoke-induced oxidative stress and subsequent downstream gene expression changes in AMs result in Bak/Bax and cytochrome c-dependent apoptosis [170] increasing the cell debris pool that needs to be removed from the lung tissue to prevent secondary inflammation.

Finally, iron metabolism is dysregulated in the lungs of patients with COPD. Iron regulatory protein 2 and hemosiderin overexpression cause cellular and mitochondrial deposition of iron in alveolar tissue and resident macrophages which is associated with neutrophilia and infective exacerbations [171, 172]. Indeed, a recent report showcased the enhanced nutrient uptake and storage in AMs from patients with COPD. Philippot et al. found that these cells present with increased transferrin and ferritin expression important for iron uptake and storage [173]. Iron-loaded AMs from smokers also secrete higher amounts of ferritin than nonsmokers [174, 175] which could catalyse oxidative stress reactions in the alveolar tissue.

It has become apparent that exacerbated oxidative stress in AMs of patients with COPD impacts on other physiological pathways. For instance, oxidation of phospholipids in AMs impairs bacterial intracellular killing in mice [176]. To date, investigation of such concepts with available analytical tools is challenging. On the contrary, whole transcriptome analysis approaches complemented by bioinformatic coexpression network analysis would allow to link the expression patterns of dysregulated oxidative stress genes to the rest of the transcriptome in order to uncover overlooked interconnected biological pathways.

### 4.3. Deregulation of the Protease-Protease Inhibitor Balance

COPD progression correlates with the persistent activation of AMs and changes in the balance of secreted proteases and protease inhibitors (Figure 2). The importance of these molecules was illustrated in an experimental model of COPD where macrophage infiltration and the expression of proinflammatory mediators were induced in response to released mast cell-tryptases [177, 178].

ECM degradation enzymes, such as MMPs and cathepsins, are produced by macrophages and result in elastolysis and alveolar tissue damage [179–182]. Furthermore, these proteases have the potential to cleave small proteins and expose chemotactic fragments or they act as chemoattractants themselves and perpetuate macrophage accumulation in the lungs [183, 184]. On the contrary, cigarette smoking has been shown to induce the functional inactivation of *α*_1_-antitrypsin, a NE inhibitor, which leaves smokers vulnerable to lung tissue destruction [185].

Monocytes and AMs are potent producers of several proteases; MMPs including MMP-1, MMP-2, MMP-7, MMP-9, and MMP-12, and cathepsins, such as K, L, B, and S, [180, 181, 184, 186, 187] and study have documented the overexpression of MMP-1, MMP-2, MMP-7, MMP-9, and MMP-12 in the lungs of smokers compared to healthy individuals [154, 188–194].

In patients with COPD, the expression of MMP-9 by AMs was shown to coincide with that of tissue inhibitor of metalloproteinases 1. The balance of these two mediators can be detrimental for the level of tissue damage in COPD lung and is controlled by the anti-inflammatory cytokine IL-10 [195]. Additional evidence for the current consensus of protease-protease inhibitor deregulation in macrophages from patients with COPD was provided by the fact that human patients with the most common *α*_1_-antitrypsin mutation have greater proteolytic activity partially due to higher expression levels of the membrane-bound serine protease matriptase [196].

Furthermore, patients with COPD have more MMP-12-positive macrophages than healthy individuals in their lungs [193]. Macrophages are the main source of MMP-12 in the lungs of emphysematous mice [113, 182], and this MMP was shown to be important for connective tissue breakdown and neutrophil recruitment [99]. The mechanism MMP-12 utilises to promote inflammation was shown to involve the cleavage of the TNF precursor on the surface of macrophages and its release to the lung microenvironment [197].

Lastly, a perhaps not so well-documented function of AMs in COPD is their chitinolytic activity. Chitinases are released in the bronchoalveolar fluid of patients and are overexpressed by AMs from patients with COPD [198]. The presence of chitinase 1 and YKL-40, a chitin-binding protein, was found to correlate with airway obstruction and emphysema and to promote the production of proinflammatory mediators, such as cytokines, chemokines, and proteases by AMs from patients with COPD [198–200]. To date, we do not fully understand whether the upregulation in the expression of chitinases by AMs is a specific immune response against fungal opportunistic infection of patients with COPD and this warrants further investigation.

Given the significance of the protease activation pathway in irreversible tissue damage, it is necessary to understand how protease and protease inhibitor production is regulated in AMs aiming to fully characterise potentially defective molecular pathways that are responsible for the imbalance in the release of these mediators. Moreover, the literature is often contradicting with regard to the identity of protease members expressed by AMs. Currently available genomic techniques could settle the discrepancy noticed between older and more recent reports and show whether genetic polymorphisms account for the deregulation of protease-protease inhibitor imbalance in AMs.

### 4.4. Impaired Pathogen Phagocytosis

Due to their strategic localisation at the host-environment interface, AMs are key players in sensing microbes and irritants and initiating the phagocytosis process in order to remove and destroy them. Macrophage phagocytosis in patients with COPD has been extensively studied in humans and animal models, and our current understanding is that AMs present with a phagocytosis defect when treated with air pollutants (Figure 2) [201, 202].

AMs from patients with COPD and cigarette smoke-treated animals have been reported to display impaired phagocytosis of pathogens, such as *H. influenzae* [203–207], *C. albicans* [208, 209], *E. coli*, *M. catarrhalis* [206, 207], and *S. pneumoniae* [205, 206, 210] compared to controls. Interestingly, defective phagocytosis of latex particles has only been described for murine AMs which implies that data generated from different species should be taken with caution [211]. It is not entirely clear whether the inability of macrophages to efficiently uptake foreign material is tissue-specific or whether it is the result of a global genetic defect. For instance, in some studies, monocyte-mediated phagocytosis was comparable with that of AMs [204], whereas in others monocytes from patients with COPD demonstrated dysregulated phagocytic abilities [212], especially when the subjects were diagnosed with acute bronchopneumonia [213]. Therefore, further work is needed to determine whether the suppressed macrophage phagocytic capacity in patients with COPD is governed by lung-specific factors.

Besides phagocytosis of external stimuli, macrophages are also responsible for the clearance of accumulating apoptotic cells to avoid the release of toxic intracellular substances which can cause secondary inflammation and inhibit tissue repair [214]. This process, coined efferocytosis, has been suggested by some studies to be compromised in AMs from patients with COPD when coincubated with apoptotic neutrophils [215, 216], eosinophils [217], or epithelial cells [150, 218, 219]. Moreover, AMs from cigarette smokers upregulate the apoptotic cell removal tyrosine kinase MERTK, arguably in a compensation mechanism to restore endogenous efferocytosis levels [220]. Interestingly, macrophage efferocytosis index was reversed in AMs from animals and patients with COPD treated with native *α*_1_-antitrypsin implying a relationship between the protease-protease inhibitor balance and apoptotic cell engulfment [221]. Moreover, mechanistic data provided by a number of groups support the idea that an increased expression of genes of the sphingosine-1 phosphate system can explain the defective efferocytic responses of AMs [222–225], although it is currently unclear whether other lipid metabolism pathways also play a role.

Studies designed to provide an insight into the molecular mechanisms that account for the suppressed AM efferocytosis showed that the expression of recognition receptors, such as CD31, CD44, CD91 [219], CR-3, CR-4, Fc*γ*R1, MARCO, and DC-SIGN, was significantly changed in AMs from patients with COPD [150, 226]. However, the expression of recognition molecules was found to be similar between smokers and patients with COPD in other reports contradicting the original findings [205]. In another report, the expression of the macrophage scavenger receptor 1 in monocyte-derived macrophages was associated with genetic variants which also controlled *in vitro* cell adhesion and survival in culture [227]. Finally, conflicting data have been published concerning the involvement of p38, ERK1/2, PI3K, ROCK, and p65 kinases and cytoskeletal changes in AM phagocytosis in COPD [147, 228].

Recently, Richens et al. showed that Rac1 activation inhibits RhoA kinase resulting in actin rearrangement and lamellipodia protrusion [229], while Minematsu et al. confirmed that RAC1 and VAV1 kinase levels are reduced in cigarette smoke-treated macrophages [216]. Therefore, it is possible that the compromised phagocytic/efferocytic capacity of macrophages in COPD can be partially explained by impaired effector kinase signalling. Finally, Bewley et al. recently showed that the defective intracellular pathogen killing exhibited by AMs from patients with COPD is caused by a MCL-1-mediated failure to increase mitochondrial ROS production [230]. Collectively, while enormous progress has been made in understanding the molecular mechanisms of altered phagocytosis in COPD, we still do not have an integrated model of the pathophysiological changes operative in AMs in this disease.

### 4.5. Surface and Intracellular Marker Expression

To date, the assessment of AM surface marker expression in patients with COPD has focused on classical M1/M2 markers [231, 232], while our own work clearly indicated that this outdated classification cannot be applied to macrophages in COPD [144]. AMs from patients with COPD express less costimulatory molecules, such as the T cell activation and survival signalling molecule CD80, major histocompatibility antigens [150, 233], Fc*γ* receptors and integrins on their surface [234], more CD163, and carbohydrate and lipid scavenger receptors, such as CD206 and CD204 than non-COPD smokers and nonsmokers [235].

Similarly, as already indicated above, the expression of surface PRRs is modulated in patients with COPD; TLR2, TLR4, and TLR5 are expressed at lower levels in macrophages from patients with COPD [126, 148, 236, 237]. However, there is contradicting evidence regarding the regulation of TLR2 expression which suggests that more work is needed to delineate whether this PRR and subsequent downstream signalling pathways play a role in the functional differences observed between macrophages from healthy individuals and patients with COPD. In contrast to the aforementioned receptors, TLR3 expression as well as downstream effector molecules, such as IL-8 and MMP-9, are overexpressed in macrophages in COPD [238]. Furthermore, polymorphisms in certain PRRs, such as TLR9, are associated with the compromised proinflammatory mediator secretion described above [206]. Lastly, patients with COPD have more CD163^+^ macrophages in their lungs [239] which is most likely the consequence of lung microenvironment imprinting, as incubation of a human macrophage cell line with sputum from patients with acute exacerbation of COPD induced the expression of other anti-inflammatory genes, such as CD206 and arginase *in vitro* [240].

## 5. IMs and Monocytes in COPD

The literature has mainly focused on the role that AMs play in COPD. However, not much is known about the functions of IMs in the lung or monocytes in the blood (Figure 2 and Table 2). In mice, inhaled smoke causes an accumulation of CX_3_CR1^+^ monocytes and lung macrophages which associate with lung inflammation [241]. Monocytes infiltrate the lung and were shown to replace the dying resident macrophages [242]. In particular, CX_3_CR1^−^GR-1^hi^ monocytes undergo a differentiation step into CX_3_CR1^+^GR-1^lo^ cells before subsequently differentiating into lung macrophages after an inflammatory insult or the depletion of lung-resident macrophages [243]. Whether this is also the case for humans remains an open question.

Monocytes are believed to develop into lung parenchyma macrophages which in mice have been identified as CX3CR1^hi^CD11b^+^CD11c^hi^MHC-II^hi^ macrophages and express TNF-*α* and IL-6 [244]. More evidence for the presence of monocytes in the human lung during inflammatory diseases came from the characterisation of a CD14^+^HLA-DR^+^ macrophage population in the sputum of patients with COPD capable to produce high levels of TNF-*α* [245]. In the lung, recruited monocytes have been shown to modulate neutrophil infiltration via the secretion of proinflammatory mediators [246].

Similar to AMs, monocyte activation in patients with COPD presents with gene expression signatures related to apoptosis, protease function, proliferation and differentiation, glycerol metabolism, and cytosolic transport as shown by a microarray study [247]. As a result of their activation state, monocytes display more prominent migration towards CCL5, CXCL1, CXCL7, or CXCR3 chemokine gradients [248, 249], production of IL-6 and CCL2, but less IL-8, MMP-9, and CD54 compared to controls [146]. In contrast, the literature on phagocytosis by monocytes from healthy individuals and patients with COPD is contradictory [205, 250]. With regard to MMP production, Pérez-Rial et al. showed that the recruited monocytes are responsible for the overall increase of macrophage numbers in a murine model of COPD [251]. Interestingly, monocyte/macrophage responses depend a lot on the causative agent of COPD as exemplified in a diesel exhaust particle-induced study where monocytes exhibited less CXCL8 and phagocytic responses due to dampened CD11b, CD14, and CD86 surface expression [252], while they overexpress CCR2 in smokers [253].

There have been various mechanistic lines of evidence to explain the augmented proinflammatory phenotype of monocytes; Dang et al., for example, found that miRNA expression, such as miR-24-3p and miR-93-5p, correlates with dysregulated downstream TLR and NOD-like receptor signalling proteins, such as I*κ*B*α* [254]. On top of that, altered epigenetic cues as exemplified by the downregulation of HDAC levels cause an upregulation in proinflammatory gene expression and NF-*κ*B-mediated inflammation [160, 255].

## 6. Concluding Remarks

COPD affects around 328 million people worldwide, and it is projected to rank within the top four most fatal diseases by 2030 [77, 256]. Moreover, the chronic nature of the disease and the frequently observed exacerbations and comorbidities have major consequences on patients' lives and countries' economic status [256]. It is therefore important to advance our knowledge of immune system manifestations in COPD and uncover the molecular pathways responsible for the cross talk between immune cells and the lung stroma in order to provide the clinic with prognosis/diagnosis biomarkers and the pharmaceutical industry with novel testable genes/pathways for future drug development screenings.

Already in 1979, it had been suggested that the macrophage population, which comprises of lung-resident macrophages and blood monocytes, constitutes more than 97% of all cells in the human bronchoalveolar lavage [257], while two decades later, the severity in COPD was linked to the presence of macrophages, neutrophils, NK cells, and activated epithelial cells in the lung [258]. However, due to the lack of specific markers and respective technologies at that time, no further subset specifications or functional subdivision could be performed and these studies remained incomplete. This is also true for studies which suggested correlation between COPD severity or small airway infiltration and macrophages [259–261] and reports which demonstrated less apoptosis and more proliferation in AMs from smokers [262]. Taken together, many of the findings concerning the role of certain immune cells and their relation to disease state, severity, and outcome have been obtained more than two decades ago. While still of value, these findings are challenged by very recent findings concerning cellular classification and function of immune cells in general.

With regard to lung macrophage populations, the efforts to better appreciate their role in COPD remain elusive. AMs are the only lung-resident macrophage population that has been extensively investigated in the past, whereas IMs have long been considered solely as an intermediate step in monocyte differentiation mainly due to limitations associated with their harvest from human subjects. The field is missing out on valuable information about potentially existing homogeneous macrophage subsets with distinct phenotypes associated with a pathological feature or clinical subgroup of COPD. In addition, the molecular mechanisms that dictate the functions of lung macrophage populations remain poorly characterised; for example, although there is evidence that the dysfunctions of lung macrophages in COPD are regulated epigenetically, an unbiased evaluation of the interplay between transcription factors and epigenetic networks active in lung macrophages in COPD is currently lacking.

**Figure 3:**
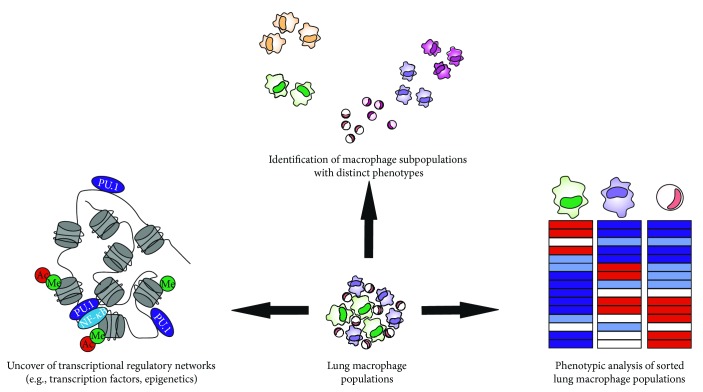
Future directions in COPD lung macrophage population research. Recent advances in Immunogenetics and Structural Biology make it possible to evaluate the heterogeneity of lung macrophage populations. In particular, single cell RNA sequencing can identify homogeneous macrophage subsets with distinct transcriptomes and functions. Mass cytometry can complement and validate initial findings establishing prognosis/diagnosis biomarkers for human patients with COPD. Moreover, analysis of the nuclear heterochromatin state with ATAC sequencing and subsequent validation with ChIP-sequencing can shed light on the epigenetic regulation of lung macrophage populations and highlight the molecular mechanisms responsible for their functions *in vivo*. Lastly, the role of AMs, IMs, and lung monocytes warrants further investigation in order to better understand the contributions of each macrophage population to COPD progression and severity. Transcriptome analysis will determine whether these populations are distinct or part of a differentiation continuum from the monocyte to the AM phenotype and will associate gene expression with unique biological processes.

To this end, latest advances in the fields of Immunogenomics and Systems Biology have been very encouraging and can help address these open questions (Figure 3). The deconvolution of the lung macrophage structure with high-dimensional single cell technologies, such as RNA sequencing, could identify lung-resident macrophage subpopulations with unique transcriptomes that reflect the niche, activation state, or interactions with other immune cells at the time of harvest [232]. Subset-specific genes could then be associated with a COPD subgroup and be validated with mass cytometry. Such an approach could stratify COPD patient cohorts according to new biomarkers and replace currently used symptom-based readouts [263].

Furthermore, the early discovery of HDAC downregulation in patients with COPD should be followed up by complementary assay for transposase-accessible chromatin (ATAC) sequencing to predict complex networks of histone-modifying enzymes and transcription factors that direct transcription in lung macrophages and link them to certain genes/biological functions [232]. Subsequent chromatin immunoprecipitation (ChIP) sequencing would validate these targets and lead to new hypothesis generation and potentially novel therapeutic interventions.

To conclude, there are many exciting research avenues to be followed, now supported by genetic and computational approaches made available in the last decade. The high level of macrophage plasticity *in vivo* implies that there are complex stimulatory and regulatory molecular circuits that act simultaneously and result in their physiological dynamics. Hence, to better understand the role lung macrophages play in COPD, we will need to take advantage of these novel tools and revisit older findings.

## Figures and Tables

**Table 1:** Molecules differentially expressed by AMs from animals or patients with COPD compared to healthy controls.

Molecule family	Encoded proteins	References
Cytokines	TNF-*α* ↓, IL-1B ↑↓, IL-6 ↓, IL-10 ↓, IL-12 ↑, Tnf-*α* ↓, Il-6 ↓	[126, 145, 147, 148, 150–152]
Chemokines	IL-8 ↓, CCL2 ↑, CCL5 ↓, CCL7 ↑, CCL13 ↑, CCL22 ↑, Cxcl10 ↓, CXCL9 ↓, CXCL10 ↓, CXCL11 ↓	[126, 145, 147–149, 151–154, 158, 165]
Chemokine receptors	CCR2 ↑, CCR5 ↑	[153, 154]
Prostaglandin metabolism	PTGS1 ↑, PTGS2 ↑	[156]
Oxidative stress	GSH ↓, Gsh ↓, iNOS ↑, HO-1 ↓	[147, 150, 155, 165, 167]
Iron metabolism	Hemosiderin ↑, *transferrin* ↑, transferrin receptor ↓, ferritin ↑	[172–175, 219]
Proteinases	MMP-1 ↑, MMP-2 ↑, MMP-7 ↑, *MMP-9* ↑ (SNPs), MMP-12 ↑, matriptase ↑	[154, 188–194, 196]
Neutrophil proteases and inhibitors	*α*_1_-Antitrypsin	[185]
Chitinolytic activity	CHIT1 ↑, YKL-40 ↑	[199, 200]
Recognition markers	CD31 ↓, CD44 ↓, CD91 ↓, CR-3 ↑, CR-4 ↑, DC-SIGN ↑, MARCO ↓	[150, 219, 226]
Cytoskeletal rearrangements	RAC1 ↓, VAV1 ↓, RhoA ↑	[216, 229]
Mitochondrial stress	MCL-1 ↑	[230]
Integrins, scavenger receptors, and adhesion molecules	CD11a ↓, CD11c ↑, CD163 ↑, CD204 ↑, CD206 ↑, MSR-1 (SNPs), MERTK ↑	[220, 227, 234, 235]
Antigen presentation molecules	MHC-I ↓, MHC-II ↓, HLA-DR ↓, CD80 ↓	[150, 233]
Fc gamma receptors, PRRs	Fc*γ*R1 ↑, CD16 ↓, TLR2 ↓, TLR3 ↓, TLR4 ↓, TLR5 ↑, TLR9 (SNPs)	[126, 148, 150, 158, 165, 206, 233, 234, 236–238]

**Table 2:** Molecules differentially expressed by monocytes or IMs from animals or patients with COPD compared to healthy controls.

Molecule family	Encoded proteins	References
Cytokines	TNF-*α* ↓, IL-6 ↑	[146, 245]
Chemokines	CCL2 ↑, IL-8 ↓	[146, 252]
Chemokine receptors	CCR2 ↑	[253]
Metalloproteinases	MMP-9 ↓, Mmp-12 ↑	[146, 251]
Antigen presentation molecules	CD86 ↓	[252]
Integrins, PRRs	CD11b ↓, CD14 ↓, CD54 ↓	[146, 252]
MicroRNAs	miR-24-3p ↑, miR-93-5p ↑, miR-320a ↑, miR-320b ↑, miR1273g-3p ↓	[254]

## Abbreviations

AM: Alveolar macrophage
ATAC: Assay for transposase-accessible chromatin
COPD: Chronic obstructive pulmonary disease
ChIP: Chromatin immunoprecipitation
DC: Dendritic cell
ECM: Extracellular matrix
GSH: Glutathione
HO-1: Heme oxygenase 1
IM: Interstitial macrophage
MMP: Metalloproteinase
NE: Neutrophil elastase
PRR: Pathogen recognition receptor
ROS: Reactive oxygen species.
